# Untypical bilateral breast cancer with peritoneal fibrosis on ^18^F-FDG PET/CT: case report and literature review

**DOI:** 10.3389/fmed.2024.1353822

**Published:** 2024-04-29

**Authors:** Lele Song, Yongkang Qiu, Wenpeng Huang, Xinyao Sun, Qi Yang, Yushuo Peng, Lei Kang

**Affiliations:** Department of Nuclear Medicine, Peking University First Hospital, Beijing, China

**Keywords:** bilateral breast cancer, peritoneal fibrosis, renal metastasis, ^
18^ F-FDG PET/CT, case report

## Abstract

**Background:**

Retroperitoneal fibrosis, a condition of uncertain origin, is rarely linked to 8% of malignant cases, including breast, lung, gastrointestinal, genitourinary, thyroid, and carcinoid. The mechanism leading to peritoneal fibrosis induced by tumors is not well understood, possibly encompassing direct infiltration of neoplastic cells or the initiation of inflammatory responses prompted by cytokines released by tumor cells. We report a case of breast cancer with renal metastasis and retroperitoneal fibrosis detected using ^18^F-FDG PET/CT, providing help for clinical diagnosis and treatment.

**Case report:**

A 49-year-old woman was referred to the hospital with elevated creatinine and oliguria for over a month. Abdominal computer tomography (CT) and magnetic resonance imaging (MRI) showed a retroperitoneal fibrosis-induced acute kidney injury (AKI) was suspected. However, a percutaneous biopsy of the kidney lesion confirmed metastasis from breast cancer. The physical examination revealed inverted nipples and an orange peel appearance on the skin of both breasts. Ultrasonography revealed bilateral hyperplasia (BIRADS 4a) of the mammary glands and bilateral neck and axillary lymphadenopathy. Subsequently, ^18^F-deoxyglucose positron emission tomography/computer tomography (^18^F-FDG PET/CT) detected abnormally high uptake (SUVmax) in the bilateral mammary glands and axillary lymph nodes, suggesting bilateral breast cancer. Furthermore, abnormal ^18^F-FDG uptake was detected in the kidney, suggesting renal metastasis. In addition, abnormal ^18^F-FDG uptake was observed in the vertebrae, accompanied by an elevation in inhomogeneous bone mineral density, raising suspicion of bone metastases. However, the possibility of myelodysplasia cannot be dismissed, and further investigations will be conducted during close follow-ups. There was significant ^18^F-FDG uptake in the retroperitoneal position indicating a potential association between retroperitoneal fibrosis and breast cancer. The final pathological diagnosis of the breast tissue confirmed bilateral invasive ductal carcinoma. The patient had been treated with 11 cycles of albumin-bound (nab)-paclitaxel (0.3 mg) and had no significant adverse reaction.

**Conclusion:**

In this case, neither the bilateral breast cancer nor the kidney metastatic lesion showed typical nodules or masses, so breast ultrasound, abdominal CT, and MRI did not suggest malignant lesions. PET/CT played an important role in detecting occult metastases and primary lesions, thereby contributing to more accurate staging, monitoring treatment responses, and prediction of prognosis in breast cancer.

## Introduction

Retroperitoneal fibrosis is an uncommon disorder of unclear etiology, with 8% of cases associated with malignancies, among which breast cancer is a relatively common cause. The pathogenesis of peritoneal fibrosis caused by tumors remains unclear, potentially involving direct neoplastic cell infiltration or inflammatory reactions triggered by cytokines secreted by tumor cells. Retroperitoneal fibrosis is characterized by increased fibrotic deposition in the retroperitoneum, frequently leading to ureteral obstruction [1]. Herein, we report a case of breast cancer with retroperitoneal fibrosis and renal insufficiency as the first symptoms. Conventional imaging did not reveal any evidence of breast cancer or distant metastases. However, ^18^F-deoxyglucose positron emission tomography/computer tomography (^18^F-FDG PET/CT) images demonstrated increased ^18^F-FDG uptake in the breast and kidney, which was suspicious of bilateral breast cancer (BBC) with diffuse renal metastatic lesions. PET/CT plays a crucial role in detecting primary lesions and metastatic lesions, monitoring relapses, managing treatment, and improving patient prognosis. In addition, we summarized the basic information and clinical details of some cases with tumor-associated retroperitoneal fibrosis in Table 1.

**Table 1:** Literature review of tumor-related retroperitoneal fibrosis.

Case	Primary tumor	Age	Authors	Sex	Clinical symptom	Management	Prognosis
1	Breast cancer (intraductal carcinoma of the breast)	73 years	Kava et al. (1)	F	Bilateral hydroureteronephrosis	Not mentioned	Not mentioned
2	Breast cancer (invasive lobular carcinoma)	73 years	MacNeil et al. (2)	F	Abdominal pain	Not mentioned	Not mentioned
3	Breast cancer (invasive lobular carcinoma)	68 years	Kane et al. (3)	F	Small bowel obstruction and hydronephrosis	Chemotherapy and operative treatments	Died
4	Breast cancer (invasive lobular carcinoma)	44 years	Kane et al. (3)	F	AKI and bilateral hydronephrosis	Not mentioned	Died
5	Breast cancer (invasive lobular carcinoma)	65 years	Gogas et al. (4)	F	Left hydronephrosis	Operative treatments	Good recovery
6	Lymphoma (follicular lymphoma)	56 years	Lan et al. (5)	F	Back pain and bilateral hydronephrosis	Chemotherapy	Complete remission
7	Lymphoma (B-cell non-Hodgkin lymphoma)	66 years	Alvarez et al. (6)		Back pain	Radiation therapy and rituximab therapy	Complete remission
8	Esophageal cancer (esophageal squamous cell carcinoma)	71 years	Mori et al. (7)	M	AKI	Chemotherapy	Died
9	Gastric cancer (gastric signet-ring-cell adenocarcinoma)	39 years	Benesch et al. (8)	M	Abdominal pain and dyspepsia	Palliative care	Died
10	Lung cancer (lung adenocarcinoma)	74 years	Nishiyama et al. (9)	F	Left lumbar backache	Chemotherapy	Died

## Case presentation

**Figure 1:**
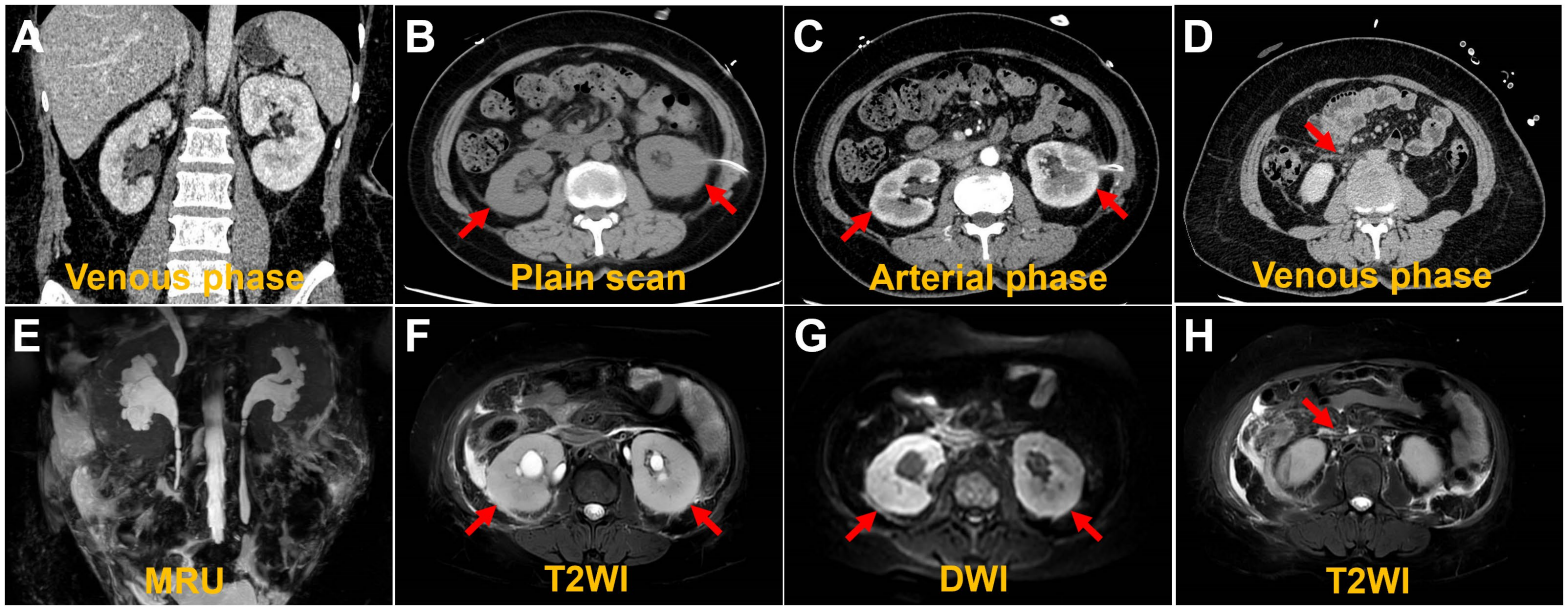
Computed tomography images and magnetic resonance images. The coronal image shows bilateral hydronephrosis and enlarged kidneys **(A,E)**. The axial images show diffuse lesions in the renal parenchyma, with no obvious masses detected **(B,C,F,G)**. The axial image shows retroperitoneal multiple exudation **(D,H)**.

A 49-year-old woman was referred to the hospital with elevated creatinine and oliguria for over a month. The patient experienced abdominal pain and lower back pain with noticeable pitting edema in both lower extremities, along with decreased urine, without an apparent trigger over a month ago. The patient has no family history of the disease. Laboratory results revealed a serum creatinine level of 1,054 μmol/L and a hemoglobin level of 76 g/L, suggestive of acute kidney injury (AKI). Ultrasonography demonstrated bilateral hydronephrosis with widening of the proximal ureters and moderately low echogenicity around the abdominal aorta, suggesting that retroperitoneal fibrosis might be present. Abdominal CT and magnetic resonance imaging (MRI) (Figure 1) revealed bilateral kidney swelling, dilation of the bilateral kidneys, renal calyces, and upper ureters, accompanied by multiple retroperitoneal and bilateral perirenal exudations, suggestive of retroperitoneal fibrosis. No focal lesions were found in either kidney.

**Figure 2:**
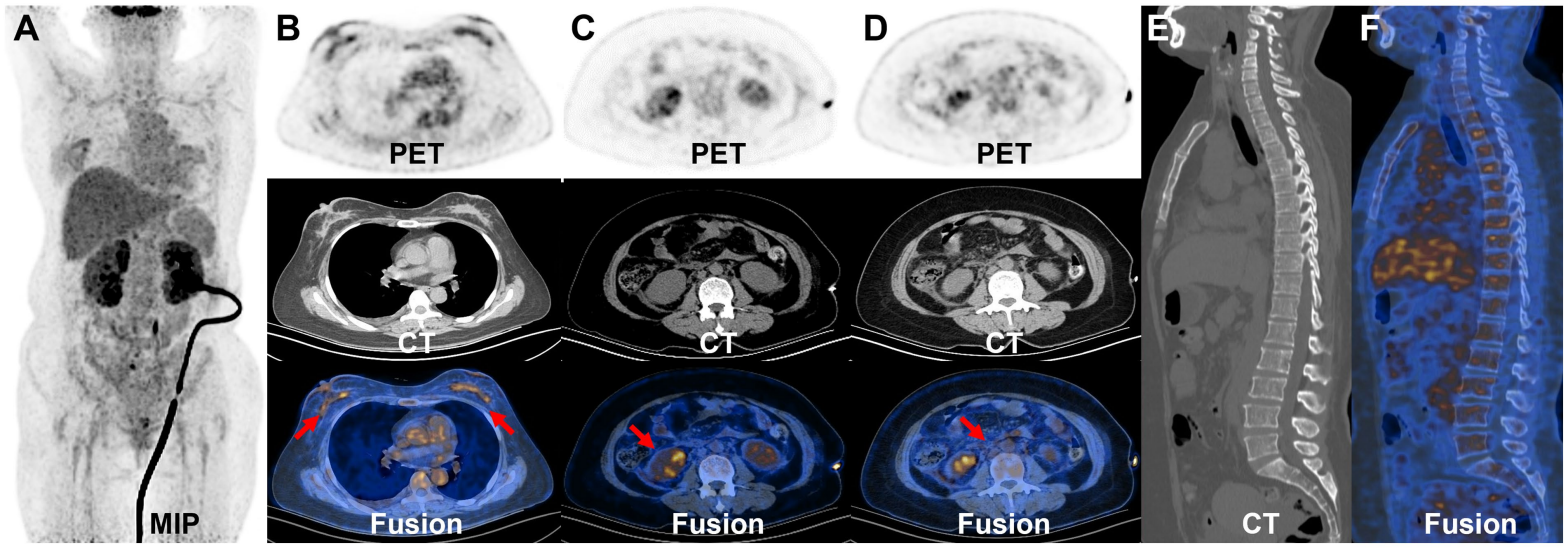
^18^F-FDG PET/CT images. The MIP demonstrated patchy metabolic elevation on both sides of the mammary glands, as well as increased volume in both kidneys, accompanied by a diffusely uneven elevated metabolic lesion **(A)**. The bilateral dense breast tissue shows diffuse mild ^18^F-FDG uptake, with an SUVmax of approximately 4.7. There is a local thickening of the skin in both breasts, with an SUVmax of 2.3, more prominent on the right side **(B)**. The transverse images show inhomogeneous ^18^F-FDG uptake in the bilaterally enlarged kidneys, with an SUVmax of approximately 8.2 **(C)**. The transverse images show right perirenal fascia thickening with a significantly higher uptake of an SUVmax of approximately 3.3 **(D)**. The sagittal images show the heterogeneous bone density elevation with radioactive distribution, SUVmax of approximately 2.9 **(E,F)**.

AKI is likely attributed to post-renal obstruction secondary to retroperitoneal fibrosis. To further define the histological features of renal lesions and exclude the probability of primary kidney disease, a biopsy was performed of the lower pole of the right kidney. The biopsy of the lower pole of the right kidney demonstrated acute renal tubular necrosis and heterotypic cells in the renal tissue. The tumor cells have moderate dysplasia, form adenoid arrangements, and show an invasive growth pattern. Immunohistochemical results showed that P120-, HER2-, PR 90%, ER 90%, E-Cadherin-, GATA (3+), CKpan (3+), and Vimentin-. Combined with the results of immunohistochemistry, it was consistent with breast cancer metastasis. Therefore, the mammary gland of the patient was further examined. A physical examination of the patient revealed an orange peel change on the skin of both breasts and a bilateral inverted nipple. Breast ultrasound showed bilateral hyperplasia (BIRADS 4a) of the mammary glands with a reduced echo. Contrast-enhanced breast MRI was precluded by the patient’s compromised renal function and aimed at averting potential adverse reactions to the gadolinium contrast agent, including those associated with nephrogenic systemic fibrosis. Consequently, whole-body ^18^F-deoxyglucose positron emission tomography/computed tomography (^18^F-FDG PET/CT) imaging was conducted to pinpoint the most reliable clinical biopsy site. Moreover, ^18^F-FDG PET/CT proves valuable in distinguishing between benign and malignant lesions, as well as in determining breast cancer staging, grading, and locating primary and metastatic tumor foci. The anteroposterior 3-dimensional maximum intensity projection (MIP) image demonstrated patchy metabolic elevation on both sides of the mammary glands, along with diffusely uneven metabolic foci in both kidneys (Figure 2A). Bilaterally dense mammary glands with a diffuse, mild ^18^F-FDG uptake were seen, with a maximum standardized uptake value (SUVmax) of 4.7. Bilateral local thickening of the breast skin with an SUVmax of 2.3 was found, which was higher on the right side. BBC was considered (Figure 2B). Enlargement of both kidneys with various degrees of ^18^F-FDG uptake is found, with an SUVmax at approximately 8.2 (Figure 2C). A patchy soft tissue density lesion is visible around the renal fascia and adjacent to the abdominal aorta, with mild ^18^F-FDG uptake, and the SUVmax is approximately 3.3 (Figure 2D). Furthermore, diffuse inhomogeneous ^18^F-FDG uptake was observed in bone lesions, characterized by an SUVmax of approximately 2.9. Additionally, inhomogeneous hyperintensities in bone mineral density were identified in the cones (Figures 2E,F), prompting suspicion of potential bone metastasis. Nevertheless, we cannot definitively exclude the possibility of myelodysplasia, and we will vigilantly monitor this concern during subsequent follow-up assessments. The lesions in the kidneys, bones, and retroperitoneal area were associated with the metastasis of breast cancer.

**Figure 3:**
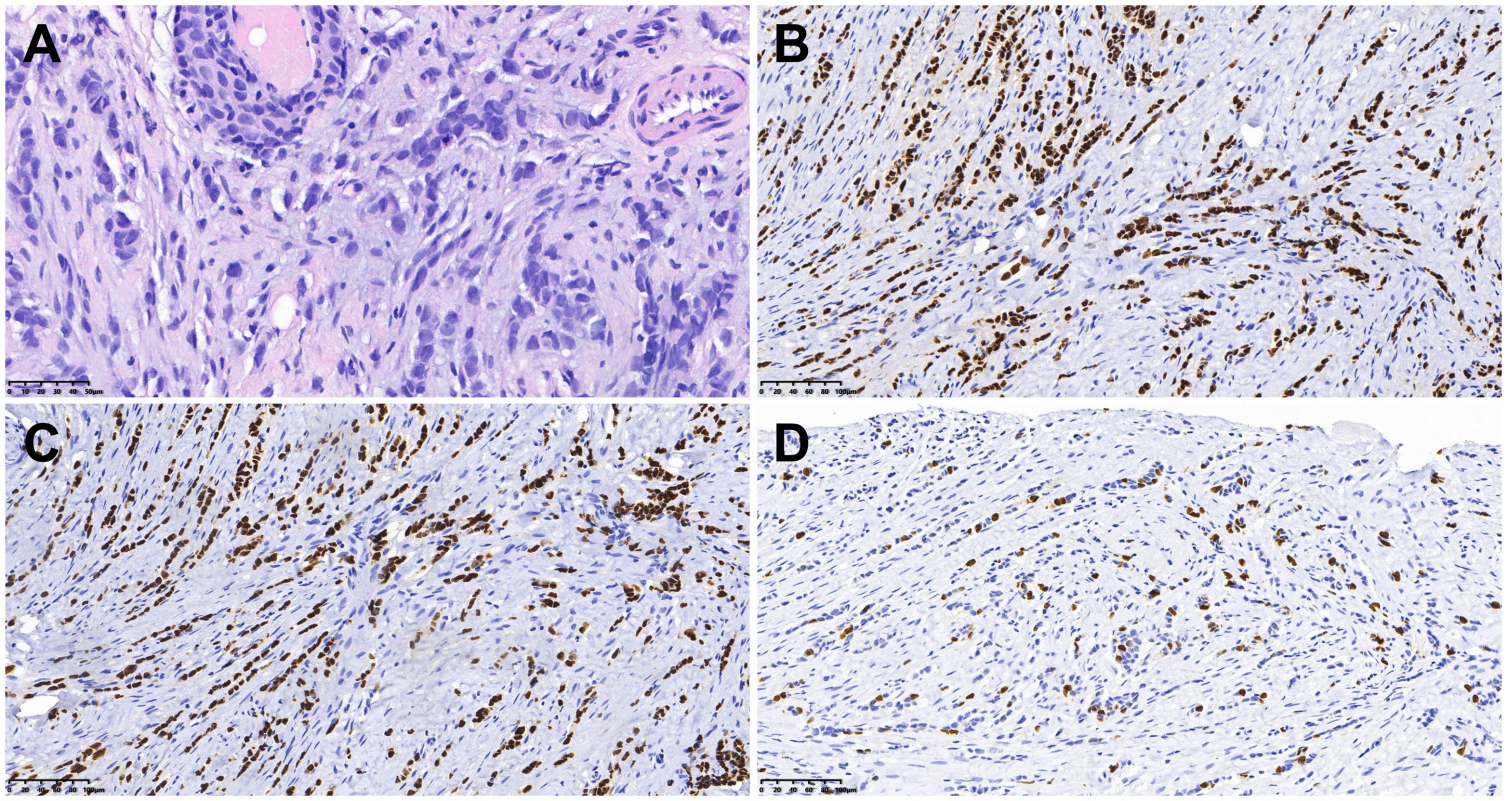
Histologic and immunohistochemical features of breast cancer. **(A)** Hematoxylin–eosin (HE) staining (×400). In immunohistochemical staining, the tumor cells were positive for ER **(B)** and GATA3 **(C)**. Moreover, 15% of them were positive for Ki-67 **(D)** [magnification **(B–D)** × 200].

Subsequently, the bilateral mammary glands were examined histologically and immunohistochemically. Pathological examination (Figure 3) revealed grade II invasive lobular carcinoma with ER 90%, PgR 50%, P40-, E-cadherin-, P120++, GATA (3+), Ki67 15%, HER2-, and PD-L1- in both breasts. The clinician diagnosed stage IV invasive breast cancer. The presence of the BRCA1 gene mutation was identified in the germline of the BBC. Renal metastases due to breast cancer were diagnosed on the basis of immunohistochemistry. The patient was ultimately identified as having BBC accompanied by metastases to the bones, kidneys, and retroperitoneal location. Additionally, AKI was observed due to retroperitoneal fibrosis. A week later, after the contraindication of chemotherapy was excluded, the patient was treated with albumin-bound (nab)-paclitaxel (0.3 mg). At present, the chemotherapy has been 11 cycles, and the patient has had no obvious adverse reactions during the course of the chemotherapy. We are still following up.

## Discussion

Breast cancer represents 24.5% of all cancer cases in women. In 2022, breast cancer will have the highest incidence and mortality in most countries (10). BBC is uncommon and accounts for 1.4–12% of all breast cancers (11). Based on the time interval between the diagnosis of bilateral malignant tumors, BBC can manifest synchronously or asynchronously (12). The primary risk factors associated with BRCA1-related breast cancer include a family history of breast cancer, younger age (under 40 years old), multifocal cancer, a history of lobular carcinoma, inherited genetic mutations such as those in the BRCA1 and BRCA2 genes, and exposure to radiation (13–16). For BRCA1 mutation carriers, the risk of breast cancer increases substantially between the ages of 30 and 50, while for women with a BRCA2 mutation, the risks are highest between ages 40 and 60 (16). BRCA-related breast cancer is characterized by a more aggressive phenotype than sporadic breast cancer, with BRCA1-related breast cancer being more frequently high grade and triple negative, and BRCA2-related breast cancer being on average a higher histological grade than sporadic cases (17). The systematic review and meta-analysis conducted by Baretta et al. (17) showed that mutation carriers have worse overall survival than BRCA-negative/sporadic cases (hazard ratio, HR 1.30, 95% CI: 1.11–1.52) and worse breast cancer-specific survival than sporadic/BRCA-negative cases among patients with stage I–III breast cancer (HR 1.45, 95% CI: 1.01–2.07). In this particular case, the patient had no other risk factors, and genetic testing revealed a mutation in the BRCA1 gene. BRCA1 mutation carriers with breast cancer may benefit from treatment with cisplatinum (18) or olaparib (19) compared to those without a mutation. These data collectively indicate the important role of BRCA mutation status with respect to treatment decisions that may impact outcomes. Genetic mutation detection plays an essential role in the clinical management of breast cancer, as it enables physicians to more accurately identify the molecular characteristics of the disease, formulate personalized treatment plans, and predict the efficacy of therapy and patient prognosis (20, 21). Family members can understand their genetic risks through genetic counseling and undergo appropriate screening and management based on the recommendations of healthcare professionals. Moreover, studies have found that the infiltrating ductal type is the most common histology in BBC (22–24). Some studies indicate that the survival rates of patients with unilateral and BBC are comparable (25, 26). While routine radiological assessments, including mammography and ultrasonography, are conducted for women presenting with suspicious breast symptoms, these methods have certain limitations in diagnosing BBC (27). In addition, contrast-enhanced MRI is forbidden for patients with renal failure. In such a situation, PET/CT, in particular, is instrumental in detecting axillary extra-nodal disease and occult metastasis in patients with locally advanced breast cancer. Its role in assessing responses to neoadjuvant or adjuvant chemotherapy is continually evolving. In the detection of suspected recurrences, it proves more effective than traditional imaging modalities (28).

Retroperitoneal fibrosis is a rare condition of diverse etiology, associated with radiotherapy, aortic aneurysms, infections, and malignancy (29). Eight percent of cases are associated with malignancies (30). Common tumors leading to retroperitoneal fibrosis include breast, lung, gastrointestinal, genitourinary, thyroid, lymphoma, sarcoma, and carcinoid (31). The pathogenic mechanism of peritoneal fibrosis induced by tumors is unknown. It may result from a desmoplastic reaction of the retroperitoneum to malignant cells, an inflammatory reaction, or cytokines secreted by the tumor (30). We have compiled a subset of cases presenting with tumor-associated retroperitoneal fibrosis, and the basic information and clinical details are summarized in Table 1. In the literature, several cases of malignant retroperitoneal fibrosis associated with breast cancer have been documented, with the majority of reported cases featuring a history of breast cancer in the patients (1, 2, 4). Obstructive uropathy is typical of retroperitoneal fibrosis, and bilateral flank pain is the most frequent clinical presentation (1, 2, 4). Our patient presented classic symptoms of bilateral flank pain and hydronephrosis. CT reveals a diffusely infiltrative soft tissue mass enveloping retroperitoneal structures, which is a typical radiological manifestation of retroperitoneal fibrosis (32). ^18^F-FDG PET/CT can be a valuable tool in evaluating the disease activity and lesion range of retroperitoneal fibrosis and monitoring the treatment of retroperitoneal fibrosis. Furthermore, as illustrated by our cases, in some patients with malignant tumors present with peritoneal fibrosis as the initial manifestation, PET/CT can assist in identifying the primary lesion and accurately staging and grading the condition.

Breast cancer metastasis to the kidneys is rarely seen (33). In one study, the incidence of metastasis of breast cancer to the kidneys was 12% (34). The mechanism of breast cancer metastasis to the kidneys can be attributed to the physiological characteristics of the kidneys. Specifically, the substantial proportion of cardiac output dedicated to renal blood flow makes it likely for circulating tumor cells to enter the renal capillaries (35). Common symptoms of renal metastasis are flank pain and hematuria (36, 37). The most common form of metastatic renal carcinomas is multiple bilaterally circumscribed, rounded masses (35). In patients with known primary tumors, diffuse enlargement of the kidney without a clear mass may suggest renal metastatic disease (38). In this case, the imaging findings were bilaterally enlarged kidneys without focal lesions. However, PET findings indicated various degrees of ^18^F-FDG uptake in the bilateral kidneys with suspicions of breast cancer metastasis to the kidney, which was agreeable with the pathological diagnosis. In PET/CT imaging, renal metastasis may manifest as small, multiple, bilateral, circular lesions with high ^18^F-FDG uptake and a diffusely invasive, large, single mass with high ^18^F-FDG uptake. By the time the primary lesion was detected by PET/CT at some sites, especially tumors prone to renal metastasis (lymphomas and carcinomas of the lung, breast, stomach, pancreas, and colon), there was a high probability of renal involvement. A biopsy for histological analysis was performed if necessary. Conducting a thorough examination of the renal cortex through ^18^F-FDG PET, coupled with a review of anatomical images such as CT and MRI results, plays a pivotal role in the identification of renal tumors.

## Conclusion

In conclusion, we present a case study detailing the ^18^F-FDG PET/CT imaging manifestations in a BBC patient with retroperitoneal fibrosis and renal metastasis. Compared with conventional imaging, PET can find systemic metastases based on the detection of primary foci, which can be used for disease staging and the evaluation of treatment efficacy.

## Data availability statement

The original contributions presented in the study are included in the article/Supplementary material, further inquiries can be directed to the corresponding author.

## Ethics statement

Written informed consent was obtained from the individual(s) for the publication of any potentially identifiable images or data included in this article. Written informed consent was obtained from the participant/patient(s) for the publication of this case report.

## Author contributions

LS: Writing – original draft, Writing – review & editing. YQ: Conceptualization, Writing – original draft. WH: Data curation, Writing – original draft. XS: Investigation, Writing – original draft. QY: Methodology, Writing – original draft. YP: Project administration, Writing – review & editing. LK: Conceptualization, Funding acquisition, Investigation, Project administration, Supervision, Writing – review & editing.
