# Supplementary material for: Untypical bilateral breast cancer with peritoneal fibrosis on 18F-FDG PET/CT: case report and literature review
Source: Front Med (Lausanne). 2024 Apr 29;11:1353822. doi: 10.3389/fmed.2024.1353822 (PMC11089181; doi:10.3389/fmed.2024.1353822)
Supplement: Supplementary file 1 [file Data_Sheet_1.PDF]

CARE case report guidelines

CARE Checklist of information to include when writing a case report

(cc) BY-NC-ND

Topic	Item	Checklist item description	Reported on Line
Title	1	The diagnosis or intervention of primary focus followed by the words "case report"	Line1
Key Words	2	2 to 5 key words that identify diagnoses or interventions in this case report, including "case report"	Line14-15
Abstract (no references)	3a	Introduction: What is unique about this case and what does it add to the scientific literature?	Line17-23
	3b	Main symptoms and/or important clinical findings	Line24-34
	3c	The main diagnoses, therapeutic interventions, and outcomes	Line34-37
	3d	Conclusion—What is the main "take-away" lesson(s) from this case?	Line38-42
Introduction	4	One or two paragraphs summarizing why this case is unique (**may include references**)	Line45-58
Patient Information	5a	De-identified patient specific information.	None
	5b	Primary concerns and symptoms of the patient.	Line60-62
	5c	Medical, family, and psycho-social history including relevant genetic information	Line63
	5d	Relevant past interventions with outcomes	None
Clinical Findings	6	Describe significant physical examination (PE) and important clinical findings.	Line78-80
Timeline	7	Historical and current information from this episode of care organized as a timeline	Line102-104
Diagnostic Assessment	8a	Diagnostic testing (such as PE, laboratory testing, imaging, surveys).	Line80-98
	8b	Diagnostic challenges (such as access to testing, financial, or cultural)	Line67-70
	8c	Diagnosis (including other diagnoses considered)	Line99-101
	8d	Prognosis (such as staging in oncology) where applicable	Line98
Therapeutic Intervention	9a	Types of therapeutic intervention (such as pharmacologic, surgical, preventive, self-care)	Line102-104
	9b	Administration of therapeutic intervention (such as dosage, strength, duration)	Line102-104
	9c	Changes in therapeutic intervention (with rationale)	Line102-104
Follow-up and Outcomes	10a	Clinician and patient-assessed outcomes (if available)	None
	10b	Important follow-up diagnostic and other test results	None
	10c	Intervention adherence and tolerability (How was this assessed?)	Line103-104
	10d	Adverse and unanticipated events	Line103-104
Discussion	11a	A scientific discussion of the strengths AND limitations associated with this case report	Line146-156
	11b	Discussion of the relevant medical literature **with references**.	Line106-156
	11c	The scientific rationale for any conclusions (including assessment of possible causes)	Line124-126
	11d	The primary "take-away" lessons of this case report (without references) in a one paragraph conclusion	Line158-161
Patient Perspective	12	The patient should share their perspective in one to two paragraphs on the treatment(s) they received	None
Informed Consent	13	Did the patient give informed consent? Please provide if requested	Yes ☑ No ☐
